# What Is the Cost of Weight Loss? An Approach to Commercial (Dry and Wet) and Homemade Diets

**DOI:** 10.3390/ani14050679

**Published:** 2024-02-21

**Authors:** Thiago Henrique Annibale Vendramini, Henrique Tobaro Macedo, Andressa Rodrigues Amaral, Rafael Vessecchi Amorim Zafalon, Adrielly Aparecida do Carmo, Cinthia Gonçalves Lenz Cesar, Pedro Henrique Marchi, Júlio Cesar de Carvalho Balieiro, Marcio Antonio Brunetto

**Affiliations:** 1 Pet Nutrology Research Center (CEPEN Pet), Department of Animal Nutrition and Production, School of Veterinary Medicine and Animal Science, University of Sao Paulo (USP), Pirassununga 13635-900, Brazil; henrique.tobaro@hotmail.com (H.T.M.); rafael.zafalon@usp.br (R.V.A.Z.); adriellycarmo@usp.br (A.A.d.C.); cinthialenz@usp.br (C.G.L.C.); pedro.henrique.marchi@usp.br (P.H.M.); balieiro@usp.br (J.C.d.C.B.); mabrunetto@usp.br (M.A.B.); 2 Veterinary Nutrology Service, Veterinary Teaching Hospital, School of Veterinary Medicine and Animal Science, University of Sao Paulo (USP), Sao Paulo 05508-270, Brazil; andressa.rodrigues.amaral@usp.br

**Keywords:** companion animal, expense, metabolic weight, prescription diet, regimen, unconventional food

## Abstract

**Simple Summary:**

In practical terms, pet owners’ lack of awareness regarding weight reduction protocols, coupled with the high costs of commercial prescription diets, often makes it difficult for them to adhere to such programs in the long term. This study addressed the costs of weight loss per kilogram of metabolic weight in dogs and cats, considering various dietary regimens. The weight reduction protocol, supervised by veterinarians, involved eight dogs and ten cats. The results indicated that the monthly and total cost per kilogram of metabolic weight was significantly lower when using commercial dry foods compared to homemade diets (*p* < 0.001), reducing costs by 40.88% and 41.01% for dogs and cats, respectively. Despite owners’ lack of awareness and associated costs, it is concluded that commercial prescription diets offer greater financial benefit for pet weight reduction protocols, emphasizing the importance of considering economic factors when implementing weight control strategies. These findings underscore the economic challenges associated to pet weight loss and emphasize the necessity for cost-effective solutions to promote sustained owner compliance.

**Abstract:**

In the context of the rising prevalence of obesity among pets, this study aimed to assess the economic aspects of weight reduction protocols for dogs and cats, considering the lack of information and the varying costs of commercial and homemade diets. The results indicated an average weekly weight loss rate of 1.02% for dogs and 0.92% for cats, with a reduction in body fat mass (*p* < 0.005). The cost analysis included an evaluation of both dry and wet commercial prescription diets as well as homemade diets. The results unveiled higher expenses associated to wet commercial diets, followed by homemade and dry commercial diets (*p* < 0.001). The study demonstrated that despite the initial investment, the long-term benefits of weight loss, including improved health and reduced financial burdens for owners, justify the expenses incurred. This comprehensive analysis provides veterinarians and pet owners with valuable insights into the economic considerations of weight reduction protocols, facilitating informed decision making and promoting pet well-being.

## 1. Introduction

In general terms, obesity is defined by the World Health Organization [1] as the abnormal or excessive accumulation of fat that can harm well-being and a healthy life. According to the more recent and specific definition for pets [2], obesity is classified as a clinical syndrome resulting from excess body fat sufficient to compromise the health and function of different organs and systems. Obesity is considered the most common nutritional and metabolic disease in veterinary medicine, and several studies have estimated the prevalence of overweight animals at between 39% and 50% [3,4,5,6,7].

Obesity may cause changes in physiological functions and disorders that decrease the quality of life and lifespan of dogs and cats [8,9,10,11,12,13,14]. The impairment of life quality is a consequence of possible orthopedic [15,16,17], cardiovascular [18,19,20,21], respiratory [8,22,23], and metabolic disorders (insulin resistance [24] and hyperlipidemia [24,25,26]).

Furthermore, in a study conducted by German et al. [27], it was observed that the success of the weight reduction protocol had positive effects on the physical and mental health of dogs. However, there are some challenges that may contribute to the failure of a weight reduction protocol, including the owner’s motivation to follow the calorie restriction protocol and the high cost of commercial prescription diets.

There is limited knowledge regarding the comprehensive costs associated with weight loss in obese dogs and cats. Therefore, grounded in an actual and supervised weight reduction protocol, our study sought estimate the expenses associated with weight loss in dogs and cats. The main objective was to compare the costs of all prescribed diets available for obese dogs and cats on the Brazilian market with homemade diets. Prices for all foods were calculated per kilogram of metabolic weight for dogs and cats. The cost analyzes conducted in this study also considered the cost per gram of total body weight and per gram of fat eliminated during weight loss, based on body composition determined by the deuterium isotope dilution method.

## 2. Materials and Methods

All experimental procedures are in accordance with the ethical principles in animal experimentation adopted by the Brazilian College of Animal Experimentation (COBEA) and approved by Ethical Principles in Animal Research adopted by Ethic Committee on Animal Use of the Grandfood Indústria e Comércio LTDA (Dourado, Brazil).

### 2.1. Location and Animals

The study was conducted by the Veterinary Nutrology Service at the Teaching Veterinary Hospital of the School of Veterinary Medicine and Animal Science of University of Sao Paulo (USP) (Sao Paulo, SP-Brazil). Eight neutered female dogs (Yorkshire, Border Collie, Teckel, Golden Retriever, Pinscher, and mixed-breeds), aged 1 to 8 years and ten neutered mixed-breed cats, five males and five females, aged 9 to 13 years were included. All animals were obese adults, with body condition score (BCS) 9, according to Laflamme [28], and body composition was determined by the deuterium isotope dilution method [29]. Only individuals whose physical and laboratory results were within the reference range or consistent with the body fat accumulation condition participated in the experiment. Thus, only obese dogs and cats, which presented a fat mass percentage higher than 30% without comorbidities of weight gain, were accepted. The obese animals were subjected to a weight reduction protocol (the objective of the weight reduction protocol for both species was to lose 20% of their initial weight). All animals of the experimental group were housed in their owner’s residence in the city of Pirassununga, SP.

### 2.2. Body Composition

Body composition was determined by the deuterium isotope dilution method. After 8 h of fasting, 0.4 g/kg of 2H_2_O was inoculated subcutaneously. Blood samples (3 mL) were collected from the jugular vein immediately before the 2H_2_O inoculation and after 2 h. These were processed for serum extraction, stored at −20 °C, and analyzed according to the methodology described by Ferrier et al. and Brunetto et al. [29,30] at the Mass Spectrometry Laboratory, Department of Medical Clinic, FMRP/USP (Ribeirão Preto, SP-Brazil). After body water quantification, total lean mass was calculated, and, by difference, fat mass was determined (expressed as a percentage). This evaluation was performed at the beginning of the study (T0) and after weight loss, to quantify the percentage of fat mass of the animals.

### 2.3. Dogs’ Weight Reduction Protocol and Experimental Design

For weight loss, obese dogs were fed with 60% of their daily maintenance energy requirements (MER) according to the NRC equation [31]. The target weight was the initial weight of each dog minus 20% [32,33]. The equation used was the following:

ERWL (energy requirement for weight loss) = 70 × (body weight − 20%)^0.75^ = Kcal/day

The daily amount of food (food A) provided to each animal was determined considering the metabolizable energy of the hypocaloric food used in the study and the ERWL of each dog. The food was offered by the owners twice a day and the amount were controlled with a measured pot provided by the veterinarian. The hypocaloric diet was standardized for all obese dogs participating in the study. The metabolizable energy (ME) of the dry diet was 2.979 kcal/g, and the macronutrient profile was 119 g/1000 kcal of crude protein, 26 g/1000 kcal of fat, 80 g/1000 kcal of nitrogen-free extract, and 50 g/1000 kcal of total dietary fiber, based on ME provided by manufacturer.

The experimental group underwent a 6-month weight reduction protocol to achieve an ideal body score condition (BCS) of 5 on a scale of 1 to 9 [28]. Body weight and BCS records were updated every fifteen days by the same veterinarian, and adjustments to the amount of food were made to maintain the weekly weight loss rate between ≥1 and ≤2% [8]. For all dogs, a minimum 20 min of exercise per day was recommended. The data of the weight reduction protocol are shown in Table 1.

### 2.4. Cats’ Weight Reduction Protocol and Experimental Design

For cats’ weight loss, the ERWL was estimated according to the NRC equation [31]:

ERWL (energy requirement for weight loss) = 85 × (body weight)^0.4^ = Kcal/day

The daily amount of food (food H) provided to each animal was determined considering the metabolizable energy of the hypocaloric food used in the study and the ERWL of each cat. The food was offered by the owners three times a day and amounts were con-trolled with a measured pot provided by the veterinarian. The hypocaloric diet was standardized for all obese cats participating in the study. The ME of the dry diet was 3.070 kcal/g as informed by manufacturer, and the macronutrient profile was 136 g/1000 kcal of crude protein, 26 g/1000 kcal of fat, 4 g/1000 kcal of non-nitrogenous extract, and 48 g/1000 kcal of total dietary fiber, based on ME provided by manufacturer.

Throughout the experimental period, cats were kept at their homes. Body weight and BCS records were updated every fifteen days by the same veterinarian, and adjustments to the amount of food were made to maintain the weekly weight loss rate between ≥0.5 and ≤1% [32] and exercise recommendations were the same as for dogs. The data of the weight reduction protocol are shown in Table 1.

The weight reduction protocol was carried out until 20% of each animal’s initial weight was lost for both species. 

To preserve the integrity of companies, commercial names were replaced by letters of the alphabet. All dry and wet prescription diets for weight loss for dogs (dry diets: Foods A, B, C, D, F, and G; wet diet: Food X) and cats (dry diets: Food H, I, J, K, L, and M; wet diet: Food Y) on the Brazilian market were selected. For the weight reduction protocol, Food A was used for dogs and Food H for cats. Based on the results from this weight loss, the costs for the other diets were estimated (B, C, D, E, F, G, X, I, J, K, L, M, and Y). The variables used to calculate the costs related to weight loss were metabolic weight, body composition, energy requirement for weight loss (ERWL), and weight loss period. In addition, prices for commercial diets (dry and wet) were obtained in the three largest Brazilian pet shop companies and an average was estimated from this information. Based on these variables, it was possible to obtain the values of daily, monthly, and total food consumption for each animal and for each commercial diet (dry and wet) and with body composition analysis by dilution of deuterium isotopes obtained, the average daily, monthly, and total weight loss costs per kilogram of metabolic metabolism per weight and average cost per gram of body weight and fat lost during the weight loss period.

To calculate the cost of weight loss with homemade diets, two diets for dogs and two diets for cats were formulated, one based on chicken meat and the other with beef. For dogs, the dietary compositions of chicken meat and beef were, respectively, 39% cooked rice; 26.5% chicken breast; 9.4% beef liver; 7.7% carrot; 7.0% green beans; 6.6% pumpkin; 3.0% vitamin and mineral supplement; 0.8% soybean oil; and 39.6% cooked rice; 24.4% beef; 10.5% beef liver; 7.6% carrot; 8.1% green beans; 7% pumpkin; 2.8% vitamin and mineral supplement, and 0.1% soybean oil. For cats, the chicken meat and beef diet compositions were, respectively, 13% cooked rice; 53% chicken heart; 15.5% beef liver; 11.6% carrot; 6.4% vitamin and mineral supplement; 0.4% soybean oil; and 19.2 cooked rice; 47.4% beef; 15.3% beef liver; 11.5% carrot; 6.4% vitamin and mineral supplement, and 0.2% soybean oil. All ingredients were elaborated in the Optimal Formula 2000 program (Optimal Informática, Campinas, Brazil) and corrected for calculated food yield using the total correction factor (FCT) adapted by Ornellas [34]. The prices of ingredients for homemade diet were obtained in three of the largest supermarket companies in the state of Sao Paulo. The vitamin and mineral supplements were obtained from the company that produces them (Complet-Biofarm, Jaboticabal, Brazil) and thus the averages were estimated. The same estimated costs for commercial diets (dry and wet) were also determined for the homemade diets.

### 2.5. Statistical Analysis

The comparisons between obese and lean dogs and cats were carried out based on a mixed linear model that considered the fixed effect of weight loss and the random effect of the animal. Assumptions of the analysis of variance models were checked using the Shapiro–Wilk test (normality of residuals) and Levine test (homogeneity of variances). Analysis of variance was conducted, and in the case of a significant F-test, the F-test itself was considered discriminatory.

Comparisons between dry, wet, and homemade diet foods were conducted based on a mixed linear model, which considered a fixed effect of food type and random effects of brand and animal. Assumptions of the analysis of variance models were checked using the Shapiro–Wilk test (normality of residuals) and Levine test (homogeneity of variances). Data that did not exhibit normal distribution were transformed using a logarithmic link function. After data transformation, analysis of variance was performed, and when significance was detected, the Tukey’s mean comparison test was adopted. All aforementioned analyses were carried out using the PROC MIXED procedure in the Statistical Analysis System software version 9.4 (SAS Institute, Cary, NC, USA). *p* < 0.05 were considered significant.

## 3. Results

### 3.1. Weight Reduction Protocol

For dogs, the weight reduction protocol resulted in an average weekly weight loss rate of 1.02% ± 0.82 and a 21.69% ± 2.18 weight reduction in 194.25 ± 28.31 days. Body fat mass decreased from 37.86 ± 4.58 to 22.10% ± 7.49 (*p* = 0.001). For cats, the weight reduction protocol resulted in a mean weekly weight loss rate of 0.92% ± 0.19 and cats achieved 20.93% ± 2.48 of weight reduction in 164.60 ± 32.53 days. Body fat mass decreased from 37.19% ± 8.25 to 30.28% ± 9.09 (*p* < 0.001). The information on the weight reduction protocols and body composition of the animals is shown, respectively, in Table 1 and Table 2.

### 3.2. Cost Analysis of Weight Loss

**Figure 1:**
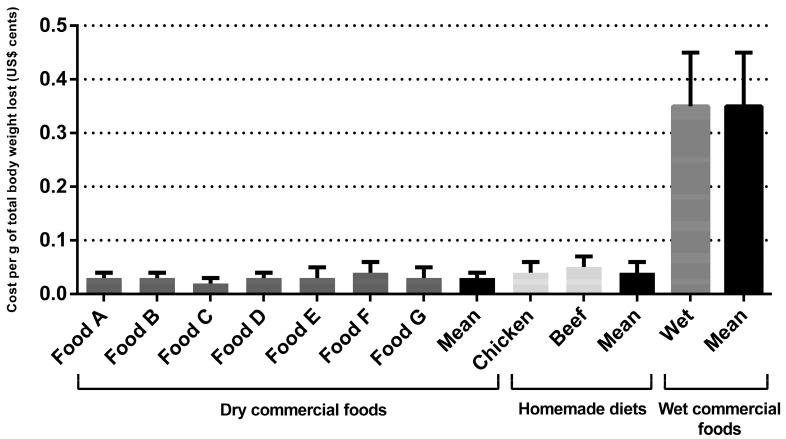
Dogs’ weight loss cost (in US$ cents) per gram of total body weight lost during the program.

**Figure 2:**
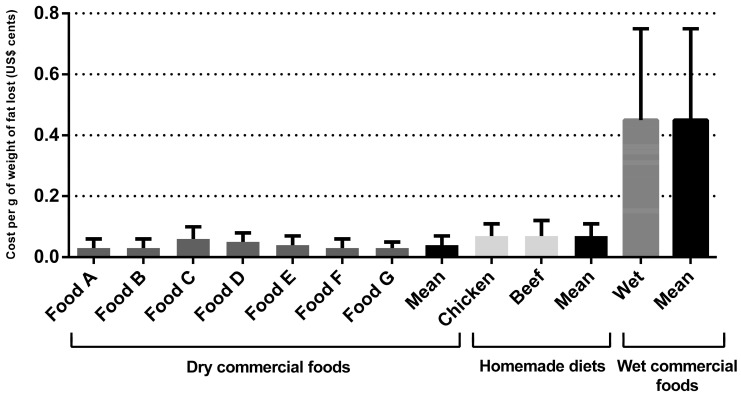
Dogs’ weight loss cost (in US$ cents) per gram of fat lost during the program.

**Figure 3:**
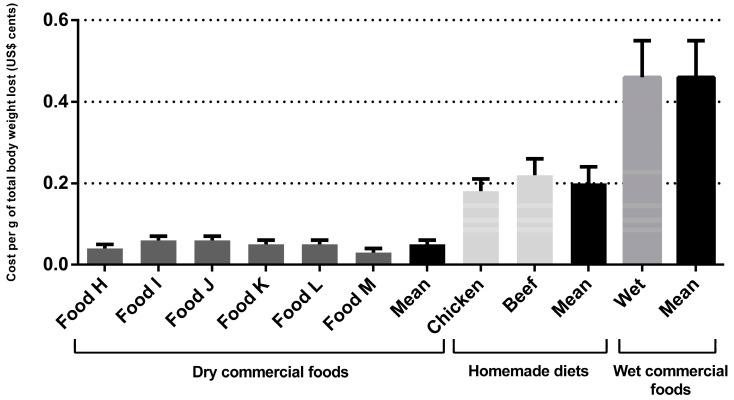
Cat’s weight loss cost (in US$ cents) per gram of total body weight lost during the program.

**Figure 4:**
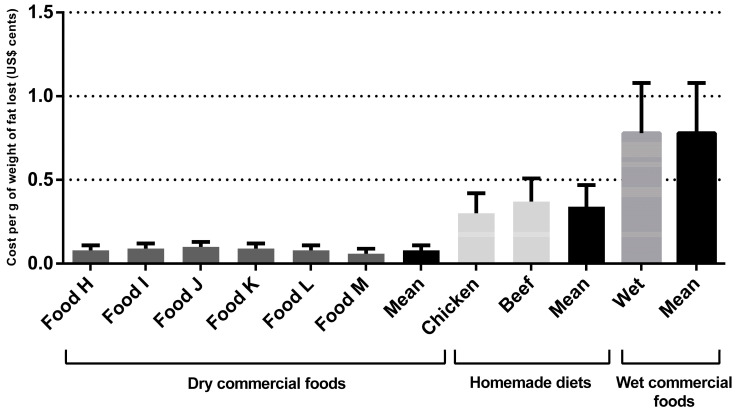
Cats’ weight loss cost (in US$ cents) per gram of fat lost during the program.

Regarding the dry commercial prescription diet for weight loss for dogs, the monthly and total cost per kilogram of metabolic weight were US$2.77 ± 0.78 and US$17.35 ± 5.66, respectively. The monthly and total cost per kilogram of metabolic weight cost for the wet commercial diet for weight loss were US$30.77 ± 3.28 and US$30.58 ± 6.19, respectively, for dogs and cats. The cost for weight loss using homemade diets was higher than dry commercial foods (*p* < 0.001) (Table 3). For the chicken-based diet, the monthly and total cost per kilogram of metabolic weight were US$4.43 ± 0.46 and US$27.68 ± 5.49, respectively. For the meat-based diet, the monthly and total cost per kilogram of metabolic weight were US$4.96 ± 0.50 and US$31.01 ± 6.16, respectively. Dry commercial diets cost an average of US$0.03 ± 0.01 cents per gram of total body weight lost, the wet commercial diet cost an average of US$0.35 ± 0.10 cents, and for the homemade diets this average was US$0.05 ± 0.01 cents (*p* < 0.001). Regarding the grams of fat eliminated, the results followed the same pattern (*p* < 0.001), with a higher cost for wet commercial diets (US$0.45 ± 0.30 cents), followed by homemade diets (US$0.07 ± 0.04 cents), compared to dry commercial prescription diets (US$0.03 ± 0.02 cents). The cost per gram of total body weight lost and per gram of fat eliminated are shown in Figure 1 and Figure 2 and Table 3, respectively. For cats, the monthly and total cost per kilogram of metabolic weight for the dry commercial diets were U$4.07 ± 0.96 and US$21.59 ± 3.66, respectively. For the wet commercial foods, these presented the highest cost (*p* < 0.001); these costs were US$30.58 ± 6.19 and US$162.16 ± 23.16, respectively, for monthly and total cost per kilogram of metabolic weight. For the chicken diet, the monthly and total cost per kilogram of metabolic weight were US$11.85 ± 3.04 and US$62.83 ± 8.97, respectively. For the meat diet, the monthly and total cost per kilogram of metabolic were US$14.78 ± 3.79 and US$78.40 ± 11.19, respectively. The dry commercial prescription diet had an average of US$0.06 ± 0.08 cents per gram of total body weight lost, while in wet commercial diets and homemade diets the averages were US$0.46 ± 0.09 and US$0.19 ± 0.04 cents (*p* < 0.001), respectively. Regarding the grams of fat eliminated, the results followed the same pattern, with a higher cost (*p* < 0.001), for wet commercial diets (US$0.78 ± 0.30 cents), followed by homemade diets (US$0.33 ± 0.13 cents) and dry commercial diets (US$0.10 ± 0.03 cents). The costs per gram of total body weight lost and per fat eliminated are shown in Figure 3 and Figure 4 and Table 3, respectively. The costs with weight loss diets are shown in Table 3. Note: all costs were estimated in Brazilian reais (R$) and then converted to US$ dollar (conversion rate: 1 US$ dollar was equivalent to R$4.91). 

## 4. Discussion

Firstly, the weight reduction protocol was followed by a reduction in fat mass, concomitantly with the maintenance of lean mass, which allows us to infer that the calorie intake, as well as protein levels in food, were adequate and guaranteed a healthy weight loss for all animals. The average weekly weight loss rate for dogs and cats was, respectively, 1.02% and 0.92% per kilogram of body weight, which is within the appropriate parameters stipulated for small animals [8,32].

The topic of obesity is currently well discussed in veterinary medicine, including the weight reduction protocol such as the recent article published with cats, by German et al. (2023) [35], evaluating weight loss in cats. However, according to Bomberg [36], there is a lack of global information on the health costs of obese and overweight animals. The information obtained in this study has the potential to provide owners with greater financial control over obesity treatment and better planning during this extended process. Despite the relatively high cost of the weight reduction protocol, weight loss proves beneficial from both a financial and health perspective in the long term. It is shown in a study that tracked 429 dogs and 372 cats over a four-year period that obese dogs’ owners spent an average of 17% more than owners of dogs in optimal body condition, and also spend 25% more on medicines [36]. As well, owners of obese cats spend 36% more on diagnosis services and 53% on surgical devices than owners of cats at ideal body weight. These data corroborate the fact that obesity is related to the incidence of diseases such as osteoarthritis, urinary incontinence, and neoplasia in dogs [10] and diabetes mellitus, neoplasia, skin diseases, oral cavity diseases, and urinary tract diseases [11] in cats.

Negative energy balance during the weight reduction protocol is based on the reduction in calories consumed by animals; to obtain the necessary energy, the animal mobilizes its fat stores with minimal loss of muscle tissue [8]. Assessing the cost per gram of weight or fat lost allows the owner to be encouraged and valued through the established weight reduction protocol.

Through the data obtained in this work, veterinarian nutritionists can provide more information that increases their versatility and the possibility of convincing the owner to adhere to the weight reduction protocol. Hence, the veterinarian can address one of the owner’s main concerns regarding the weight reduction protocol, which is, specifically, the anticipated cost of the process (refer to Table 3), determined based on the animal’s metabolic weight. However, it is important to highlight that food prices (dry commercial, wet commercial, and homemade diets) are subject to changes, which absolutely requires periodic updating of data.

The results presented in the form of kilograms of metabolic weight also allow estimating costs for any animal, since having the animal’s body weight, the metabolic weight can be calculated (kilogram of body weigh^0.75^) and multiplied by cost per unit, which estimates the outcome for the animal.

According to the data obtained in this study, it was found that, for each species, the highest cost for each variable (total, monthly, gram of total body weight lost, and gram of fat lost) were those of the wet commercial diet, followed by the homemade diet, and then the dry commercial diet. For dogs and cats, the total cost of the weight reduction protocol per kilogram of metabolic weight was higher for cats compared to dogs when using the dry commercial and homemade diet, and higher for dogs when using the commercial wet diet.

The higher costs of the wet commercial diet can be explained due to its low metabolizable energy [37], which increases the kilograms of food that need to be offered to attain the ERWL. The general higher costs for cats compared to dogs can be explained due to their higher protein requirement [31]. According to Pedrinelli et al. [38], the ingredients used in the formulation of homemade diets are generally the same as those used in human food. In addition, there is a need for owners to adapt to the homemade diet, which can lead to the removal, substitution, or changes of ingredients due to the high cost [39] and also result in nutritional deficiency [39,40].

As demonstrated by Carciofi [41], protein ingredients used in commercial dry diet formulations increase the cost of the final product, especially when ingredients with high digestibility and better amino acid profile are used. In this study, all commercial prescription dry diets for weight loss had a higher protein content and yet had lower cost. In addition, increasing the levels of this macronutrient in the homemade diet would make the formulation even more expensive and the use of this macronutrient unaffordable when the goal is to conduct a weight reduction protocol. Another concerning issue in relation to homemade diets is the use of vitamin and mineral supplements. This is one of the most expensive ingredients in the formulation and therefore many owners tend to neglect the relevance of these ingredients and do not add them correctly to the diet. It is important to highlight that this ingredient is essential to meet the essential nutrients requirement and that, without its inclusion, the diet provided to the animal will not be complete or balanced and the animal will be malnourished throughout weight reduction protocol or as long as it does not receive a complete and balanced diet.

In addition to the high cost, according to Michel [42], many other nutrients or dietary supplements have been proposed and included in dry commercial prescription diets for weight loss purposes. These include L-carnitine, a metabolite involved in mitochondrial fat transport that can increase the rate of weight loss by promoting retention of lean body mass in companion animals during calorie restriction [43]. Besides being more affordable than homemade diets, most dry commercial prescription diets contain nutraceuticals. If these nutraceuticals were included in homemade diets, the costs would further increase.

## 5. Conclusions

In conclusion, considering the costs of the diets used at the time of this study, a weight reduction protocol involves several variables, and cost values can only be accurately calculated based on a real weight reduction protocol. For both species, the highest cost for each variable (total, monthly, gram of total body weight lost, and gram of fat lost) were associated with the wet commercial diet, followed by the homemade diet, and then the dry commercial diet. Regarding dogs and cats, the total cost of the weight reduction protocol per kilogram of metabolic weight was higher for cats compared to dogs when used the dry commercial and homemade diet. However, for dogs, it was higher when using the wet commercial diet. The monthly cost of the weight reduction protocol per kilogram of metabolic weight and the cost per gram of fat and per gram of total body weight lost were higher for cats compared to dogs across all three types of diets. Finally, the cost-based calculation (US$ dollar) per kilogram of metabolic weight allows for extrapolation of the results to other dogs and cats.

## Figures and Tables

**Table 1:** Summary of weight reduction protocol and study animals (mean ± standard deviation).

Variable	Before Weight Loss	After Weight Loss	*p*
Dogs			
Number of animals	8	8	-
Body weight (kg)	22.81 ± 14.40	18.04 ± 11.22	0.003
Body condition score	9.00 ± 0.00	5.75 ± 0.46	<0.001
Total body weight loss (%)	-	21.69 ± 2.18	-
Weekly weight loss rate (%)	-	1.02 ± 0.82	-
Weight loss period (d)	-	194.25 ± 28.31	-
Cats			
Number of animals	10	10	-
Body weight (kg)	5.09 ± 0.86	4.03 ± 0.69	<0.001
Body condition score	9.00 ± 0.00	5.70 ± 0.82	<0.001
Total body weight loss (%)	-	20.93 ± 2.48	-
Weekly weight loss rate (%)	-	0.92 ± 0.19	-
Weight loss period (d)	-	164.60 ± 32.53	-

**Table 2:** Body composition determined by deuterium isotopes (mean ± standard deviation).

Variable	Before Weight Loss	After Weight Loss	*p*
Dogs			
Fat mass (kg)	8.80 ± 5.59	4.11 ± 2.71	0.005
Lean mass (kg)	14.28 ± 9.19	13.92 ± 8.80	0.538
Fat mass (%)	37.86 ± 4.58	22.10 ± 7.49	0.001
Lean mass (%)	61.14 ± 4.58	77.90 ± 7.49	0.001
Cats			
Fat mass (kg)	1.91 ± 0.58	1.22 ± 0.41	<0.001
Lean mass (kg)	3.18 ± 0.58	2.81 ± 0.62	<0.001
Fat mass (%)	37.19 ± 8.25	30.28 ± 9.09	<0.001
Lean mass (%)	62.81 ± 8.25	69.72 ± 9.09	<0.001

**Table 3:** Costs related to the weight reduction protocol (mean ± standard deviation).

Item	Group			*p*
	Dry Commercial Diet	Homemade Diet	Wet Commercial Diet	
Dogs				
Total Cost per Kilogram of MW ^1^ (US$)	17.35 ± 5.66 ^C^	29.34 ± 5.90 ^B^	192.15 ± 41.57 ^A^	<0.001
Monthly Cost per Kilogram of MW ^1^ (US$)	2.77 ± 0.78 ^C^	4.70 ± 0.55 ^B^	30.77 ± 3.28 ^A^	<0.001
Cost per gram of total body weight lost (US$)	0.03 ± 0.01 ^C^	0.05 ± 0.01 ^B^	0.35 ± 0.10 ^A^	<0.001
Cost per gram of fat lost (US$)	0.03 ± 0.02 ^C^	0.07 ± 0.04 ^B^	0.45 ± 0.30 ^A^	<0.001
Cats				
Total Cost per Kilogram of MW ^1^ (US$)	21.59 ± 3.66 ^C^	70.62 ± 12.69 ^B^	162.16 ± 23.16 ^A^	<0.001
Monthly Cost per Kilogram of MW ^1^ (US$)	4.07 ± 0.96 ^C^	13.31 ± 3.67 ^B^	30.58 ± 6.19 ^A^	<0.001
Cost per gram of total body weight lost (US$)	0.06 ± 0.08 ^C^	0.19 ± 0.04 ^B^	0.46 ± 0.09 ^A^	<0.001
Cost per gram of fat lost (US$)	0.10 ± 0.03 ^C^	0.33 ± 0.13 ^B^	0.78 ± 0.30 ^A^	<0.001

^1^ Metabolic Weight (dogs—body weight^0.75^; cats—body weight^0.67^). ^A–C^ Averages in the same line followed by different letters differed by 1% in the Tukey test adjusted by PROC MIXED.

## Data Availability

The data presented in this study are available on request from the corresponding author. The data are not publicly available due to the participants in this study did not give consent.
